# Carbon-Based Polymer Nanocomposites for High-Performance Applications II

**DOI:** 10.3390/polym14050870

**Published:** 2022-02-23

**Authors:** Ana M. Díez-Pascual

**Affiliations:** Universidad de Alcalá, Facultad de Ciencias, Departamento de Química Analítica, Química Física e Ingeniería Química, Ctra. Madrid-Barcelona, Km. 33.6, 28805 Alcalá de Henares, Madrid, Spain; am.diez@uah.es

In the field of science and technology, carbon-based nanomaterials, such as carbon nanotubes (CNTs), graphene, graphene oxide, graphene quantum dots (GQDs), fullerenes, and so forth, are becoming very attractive for a wide number of applications. For instance, in the field of biological and biomedical applications, they are widely used in biosensing, drug delivery, tissue engineering, imaging, diagnosis, and cancer therapy. In 1991, Iijima observed, for the first time, the formation of multiwalled carbon nanotubes from carbon arc discharge [1]. Afterward, research on CNTs proliferated quickly as a consequence of their unique mechanical, electrical, and structural diversity, which endows them with superior strength, flexibility, and electrical conductivity. However, among the various allotropes of carbon, graphene is regarded as the most attractive material owing to its unique intrinsic properties. The term “graphene” was initially introduced by Mouras and co-workers in 1987, who coined the term “graphitic intercalation compounds (GIC)” [2]. Over the last two decades, research on graphene has greatly increased, and numerous exceptional properties have been reported. Its oxidized form, graphene oxide, shows easy functionalization ability and aqueous processability, making it suitable for large-scale applications [3]. Other attractive nanomaterials from the carbon family are GQDs, which have excellent photoluminescence due to their quantum confinement effect, superior biocompatibility, and resistance to photo-bleaching [4]. The combination of carbon nanomaterials with polymeric matrices leads to novel nanocomposites with better performance due to synergistic effects [5,6,7,8,9], with applications in areas such as electronics, energy storage, photocatalysis, automobiles, aerospace engineering, and so forth.

This Special Issue offers selected examples regarding carbon nanomaterial-reinforced polymeric composites for a variety of these applications. Organic thermoelectric materials, such as polyaniline, poly(3-hexylthiophene) (P3HT), and poly(3,4-ethylenedioxythiophene) (PEDOT), have attracted extensive attention due to their low cost, flexibility, and inherently low thermal conductivity [10,11,12]. However, their thermoelectric factor is low compared to their inorganic counterparts. Thus, the nanocomposite strategy has been utilized to tune the thermoelectric properties of conducting polymers based on the expectation of combining the unique characters of individual nano-inclusions and/or introducing possible synergistic effects [13]. Single-walled carbon nanotubes (SWCNTs) have been extensively used as nanofillers for enhancing the thermoelectric properties of polymers, due to their outstanding electronic transport properties. However, raw SWCNTs are mixtures of semiconducting and metallic CNTs, which makes the tuning of properties difficult. In a recent work [14], pure SWCNT powders were obtained by sorting them from raw mixed SWCNTs through a simple solution centrifugation process, using regioregular poly(3-dodecylthiophene) (rr-P3DDT) as the dispersant. The SWCNT/rr-P3DDT composite films with different SWCNT contents of up to 50 wt% were prepared via direct solution casting, showing significantly enhanced Seebeck coefficients and power factors. This work shows the effectiveness of pure semiconductor SWCNTs as fillers to optimize the thermoelectric properties of polymer/CNT nanocomposites. 

However, the major challenges in utilizing CNTs include the poor interfacial bonding between CNTs and the polymer matrix, the poor dispersion of the CNTs within the polymer matrix, the improper alignment of the CNTs, and the degradation of the CNTs due to processing. As a result, the predicted properties of the CNTs could not be fully reached in modified polymer nanocomposites. To solve this issue, numerous surface modification methods for improving the quality of the filler–matrix bonding have been reported [15,16,17,18]. In particular, acid treatment using a H_2_SO_4_/HNO_3_ mixture and silane functionalization is an effective method to enhance the dispersion of the MWCNTs within kenaf and hybrid kenaf/glass fiber-reinforced epoxy multifunctional composites [19]. Thus, the compressive modulus and strength increased by 73% and 20%, respectively, for composites incorporating 1.0 wt% SWCNT compared to binary composites. 

Piezoelectric polymers, such as polyvinylidene (PVDF), with advantages such as simple processing routes, light, and thin structures, durability, high chemical resistance, low environmental sensitivity, and biocompatibility, have significant potential in wearable devices. The incorporation of SWCNTs and MWCNTs has been reported as an efficient route to induce a higher proportion of the β-phase structure and improve the overall piezoelectricity and mechanical strength of the polymer [20]. In a recent study, 0D carbon black 1D CNT–COOH and 2D GO were added to poly(vinylidenefluoride-co-trifluoroethylene (PVDF-TrFE) films to improve their crystallinity and piezoelectric effect [21]. The addition of 1 wt% CNT–COOH and polymeric dispersants increased the β-phase content in PVDF-TrFE from 74% to 86%, which in turn raised the piezoelectric coefficient by about 32%, and these films show great potential as seismocardiographic sensors, pressure sensors, and energy-harvesting elements. Flexible and wearable electronics have enormous potential in applications in human motion detection, human–computer interaction, and context identification, which have promoted the rapid development of flexible sensors. A novel work developed a facile solvent-free method toward creating a flexible pressure and stretch sensor based on a hierarchical layer of graphene nanoplates. The resulting sensor exhibited excellent pressure and strain-sensing characteristics, with a low strain detection limit at 0.1%, a large strain gauge factor of up to 36, and outstanding cyclic stability, withstanding more than 1000 cycles. Moreover, the sensor has an extraordinary pressure range as large as 700 kPa. Compared to most of the reported graphene-based sensors, this work uses a completely environmentally friendly method without any organic solvents [22].

In another work, a high-performance copolymer matrix made from Kevlar^®^- and Nomex^®^-derived chains was modified by introducing a few kinks in the linear Kevlar intractable chain to improve its solubility, and hence processability, without affecting its outstanding thermo-mechanical performance [23]. The matrix was then reinforced with exfoliated pristine graphene and graphene oxide. The carboxyl groups on the surface of the graphene oxide were chemically bonded with the amine-terminated copolymer chains, resulting in improved mechanical properties and glass transition temperatures. This novel strategy is highly suitable for tailoring the properties of high molecular weight polymers. 

GO shows improved solubility in aqueous media. However, it is insoluble in non-polar and polar aprotic solvents, which limits some applications. In this regard, different functionalization approaches have been carried out, such as the reaction with hexamethylene diisocyanate (HDI) [24]. Another approach is the functionalization with macromolecular compounds, such as poly(5-amino-1-naphthol) (P5A1N), which can be carried out by the electrochemical polymerization of 5-amino-1-naphthol (5A1N) in the presence of HClO_4_ and H_4_SiW_12_O_40_ on the surface of a Au electrode covered with the GO sheets [25]. The functionalized nanomaterial was able to support 1,4-phenylene diisothiocyanate (PDITC), a compound used as a cross-linking agent for various biological application platforms for the detection of cancer biomarkers. Overall, we hope that this issue will be useful to an interdisciplinary readership, including researchers in academia and industry in the fields of materials science, engineering, chemistry, energy, and biomedical engineering.
